# Observational study of therapeutic bronchoscopy in critical hypoxaemic ventilated patients with COVID-19 at Mediclinic Midstream Private Hospital in Pretoria, South Africa

**DOI:** 10.7196/AJTCCM.2020.v26i4.119

**Published:** 2020-10-13

**Authors:** EM Taban, GA Richards

**Affiliations:** ^1^ Critical Care Unit, Mediclinic Midstream Critical Care Unit, Private Hospital Pretoria, South Africa; ^2^ Department of Critical Care and Pulmonology, Faculty of Health Sciences, University of the Witwatersrand, Johannesburg, South Africa

**Keywords:** covid-19, coronavirus

## Abstract

Background. Flexible fibreoptic bronchoscopy (FFB) has been used for years as a diagnostic and therapeutic adjunct for the diagnosis of potential airway obstruction as a cause of acute respiratory failure or in the management of hypoxaemia ventilated patients. In these circumstances, it is useful to evaluate airway patency or airway damage and for the management of atelectasis. Objectives. To evaluate the use of FFB as a rescue therapy in mechanically ventilated patients with severe hypoxaemic respiratory failure caused by COVID-19. Methods. We enrolled 14 patients with severe and laboratory confirmed COVID-19 who were admitted at Mediclinic Midstream Private Hospital intensive care unit in Pretoria, South Africa, in July 2020. Results. FFB demonstrated the presence of extensive mucus plugging in 64% (n=9/14) of patients after an average of 7.7 days of mechanical ventilation. Oxygenation improved significantly in these patients following FFB despite profound procedural hypoxaemia. Conclusions. Patients with severe COVID-19 pneumonia who have persistent hypoxaemia despite the resolution of inflammatory parameters may respond to FFB with removal of mucus plugs. We propose consideration of an additional pathophysiological acute phenotype of respiratory failure, the mucus type (M-type).

*Afr J Thoracic Crit Care Med* 2020;26(4):Published online 12 October 2020. https://doi.org/10.7196/AJTCCM.2020.v26i4.119

Severe acute respiratory syndrome coronavirus-2 (SARS-CoV-2) is the novel coronavirus which causes COVID-19. At the time of writing (24th August 2020) and since its initial detection, more than 23 605 542 cases have been confirmed and 812 757 people have died worldwide.[1] In South Africa, the number of confirmed cases has continued to rise and is currently standing at 609 773 with 13 059 deaths since the first cases were reported in March 2020.[1] Eighty-one percent of patients with COVID-19 are asymptomatic while 14.1% present with severe disease and 4% are critically ill and require mechanical ventilation.[2]

However, despite the continued global rise in mortality since the outbreak of SARS-CoV-2 in Wuhan, China in 2019, the highly infectious nature of the virus has resulted in limited use of bronchoscopy. It is being utilised primarily for diagnostic or management purposes in non-COVID-19 patients.[3]

COVID-19 patients who require mechanical ventilation have been classified into two phenotypes according to Gattinoni[4] and these have been incorporated into the Surviving Sepsis Guideline: the L- and H-type. The L-type is characterised by low elastance (high compliance), is easy to ventilate, has low lung recruitability and may respond to early proning. The H-type is characterised by high elastance (low compliance) that resembles more closely patients with typical acute respiratory distress syndrome (ARDS) and is potentially recruitable.[5] The H-type may have a higher mortality with most patients requiring further interventions such as proning, airway pressure release ventilation (APRV) or even extracorporeal membrane oxygenation (ECMO).[6] The L-type theoretically can progress to the H type over time. Some of these patients in the L- or H- categories fail to improve their oxygenation despite optimal chemotherapy and mechanical ventilation. These patients have a prolonged ventilatory course, often complicated by secondary hospital-acquired sepsis with an associated high mortality.[7] It has been presumed that this represents a combination of irreversible pulmonary fibrosis and microvascular pulmonary thrombosis.[8]

Currently, there are no studies to support the use of flexible fibreoptic bronchoscopy (FFB) as a therapeutic tool in these patients primarily because there is no obvious evidence of atelectasis or dynamic hyperinflation suggesting airway pathology. We nevertheless decided to perform FFB after the point of maximal care had been reached without improvement in oxygenation to assess the status of the airways and to see whether there would be an impact on oxygenation.

## Methods

### Study population, setting and data collection

We enrolled patients with laboratory confirmed SARS-CoV-2 infection who were admitted to the intensive care unit (ICU) at Mediclinic Midstream Private Hospital (MMPH) in Pretoria, South Africa, on 24th July 2020 until 4th August 2020. These patients had severe COVID-19 pneumonia with the following characteristics: severe refractory hypoxaemia despite maximal mechanical ventilatory support, including proning and significant deterioration from previous minimal ventilator settings.

Maximal ventilatory settings were defined using a volume synchronised intermittent mandatory ventilation (SIMV) mode with a peak pressure >30 $cmH_2O$, a fraction of inspired oxygen ($FiO_2$) of 1.0, oxygen saturation <90%, respiratory rate ≥36 breaths/min, inspiratory: expiratory ratio of 1:1, partial pressure of oxygen in arterial blood ($PaO_2$) <60 mmHg and no worsening of radiological features or evidence of mucus plugs. In the first seven patients, the oxygenation index (OI) was not measured utilising the $PaO_2$ and arterial saturation while on the same ventilatory parameters were used instead. In the remaining seven patients, 2 had an OI >40 indicating severe pulmonary compromise, 3 had an OI in the moderate range (25 - 40) and 2 had an OI in the mild range. All laboratory tests and radiological assessments including plain chest radiography and computed tomography (CT) scan of the chest were performed at the discretion of the treating physician.

Patients 1 and 8 had been airlifted from a peripheral hospital with oxygen saturations of 74% and 88% after having been ventilated on APRV mode for 8 and 7 days, respectively. During this time, there was no improvement in oxygenation and they were subsequently referred to MMPH for consideration for ECMO therapy and further management. Four of the other patients were transferred from a peripheral hospital to MMPH for pulmonology opinion after having been on mechanical ventilation for 2 to 4 days and the remainder of the patients were *de novo* admissions to MMPH. All patients underwent high resolution CT scanning which confirmed features of severe COVID-19 pneumonia according to the British Society of Thoracic Imaging recommendation.[9] All patients were receiving ventilatory support either with APRV or lung protective, low tidal volume and SIMV mode. Patients were proned during admission, either before ventilation (the *de novo* admissions) or during ventilatory support. Eight patients received antibiotics but the remaining six patients had stopped taking antibiotics for more than 3 days prior to bronchoscopy. Plain chest X-ray and an arterial blood gas were performed 1 hour before and 2 hours after bronchoscopy. Ethics approval (ref. no. M2008102) was obtained from the University of the Witwatersrand, Johannesburg. Informed consent for both the bronchoscopy and study participation was given by the next of kin.

### Bronchoscopy procedure

The bronchoscopy was performed by a single pulmonologist while the patient was undergoing mechanical ventilation in a negative pressure room in the general ICU with all staff in full PPE (N95 masks, goggles, sterile gowns, face shields, double sterile gloves, head and shoe caps). The procedure was performed under general anaesthesia by an experienced anaesthetist.

## Results

The study had initially enrolled 16 patients with severe COVID-19 pneumonia that was complicated by ARDS and who had undergone a bronchoscopy. However, 2 patients were excluded because 1 had a loculated effusion and the other had nosocomial fungal pneumonia. The remaining patients consisted of 9 males and 5 females (Table 1).

More than 70% (*n*=10/14) of the patients were obese and 21% (*n*=3/14) were overweight (Table 1). Of the males, 11% (*n*=1/9) had class 3 obesity, 33% (*n*=3/9) had class 2 obesity and 22% (*n*=2/9) had class 1 obesity (Table 2). Of the females, 40% (*n*=2/5) had class 3 obesity, 20% (*n*=1/5) had class 1 obesity and 20% (*n*=1/5) were overweight (Table 1). The remaining female was postpartum at 42 years of age. She had delivered a live infant weighing 1.25 kg at 29 weeks of gestation by caesarean section and although the initial APGAR score was low, the condition of the infant subsequently

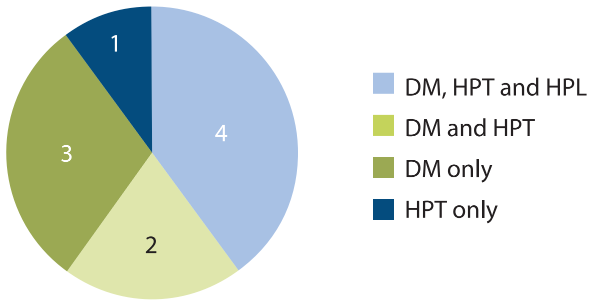

*Fig. 1. Graph showing the comorbidities of patients.*

**Table 1. The demographics of patients**

| Patient number | Sex | Age | Race | BMI | DM | HPT | Hyperlipidaemia |
|---|---|---|---|---|---|---|---|
| 1 | M | 76 | W | 24 | Yes | Yes | Yes |
| 2 | M | 48 | B | 38 | Yes | | |
| 3 | M | 74 | I | 31 | Yes | | |
| 4 | F | 67 | B | 46 | | | |
| 5 | M | 51 | W | 41 | Yes | | |
| 6 | F | 42 | B | 34 | | | |
| 7 | M | 59 | B | 36 | Yes | Yes | |
| 8 | M | 64 | B | 30 | | Yes | |
| 9 | M | 57 | W | 29 | | | |
| 10 | F | 66 | B | 40 | Yes | Yes | Yes |
| 11 | M | 48 | B | 36 | Yes | Yes | Yes |
| 12 | F | 64 | B | 33 | Yes | Yes | Yes |
| 13 | F | 69 | W | 26 | Yes | Yes | |
| 14 | M | 43 | B | 26 | | | |

BMI = body mass index; DM; diabetes mellitus; HPT = hyperparathyroidism; M = male; F = female; B = black; W = white; I = Indian.

improved. The delivery was performed prior to, but not because of, the bronchoscopy. More than a quarter of the patients (*n*=4/14) had a combination of diabetes, hypertension and hyperlipidaemia, 1 had epilepsy, 2 had hypertension and diabetes, 3 had diabetes alone and 1 had hypertension alone. Half of the patients (*n*=7/14) were older than 60 years and 28% (*n*=4/14) had no known comorbidities (Fig. 1).

The CT scan of the chest confirmed pneumonic changes consistent with a severe COVID-19 pneumonia in all the patients. Figs 2 and 3 show the CT scans of patient 1 and 2 on admission, confirming the diagnosis.

Despite no evidence of mucus plugging or atelectasis on the chest radiograph, significant mucus impaction was found during the FFB. The X-rays of patients 1 and 3 pre- and post-bronchoscopy are shown in Figs 4 and 5.

Patients 2, 5 and 9 underwent bronchoscopy immediately after intubation and no evidence of mucus plug formation was observed but repeat bronchoscopy was performed after ~ 1.75 days.

All the patients improved their $PaO_2$ and oxygen saturation and patients 8 - 14 improved both $PaO_2$ and OI after bronchoscopy (Table 2). Patients 2 and 4 showed the presence of thick gelatinous mucus within the 2nd and 3rd generation bronchi and removal of the mucus was associated with improvement in hypoxaemia despite no alteration of the mechanical ventilator settings. Patients 1, 3, 10 and 11 underwent emergency FFB after an average of 7.5 days on mechanical ventilation after having desaturated significantly with an $FiO_2$ of 1.0 without alteration of ventilatory parameters and no X-ray changes that could explain this deterioration (Table 2). Thick mucus plugs causing partial obstruction of both the main and smaller bronchi were visualised. A significant improvement in the $PaO_2$ occurred in these patients after the removal of the mucus plugs (Table 2). Half of the patients (*n*=7/14) underwent bronchoscopy after day 7 of ventilation, which also showed the presence of gelatinous mucus and partial blockage of the endotracheal tube.

The diameter of the working channel of the bronchoscope used was 2 mm, but in view of the tenacity of the mucus, biopsy forceps had to be used to facilitate extraction. Patients 1, 11 and 3 desaturated multiple times during the procedure, requiring manual bagging with a bag valve device and even required re-intubation as there was difficulty extracting

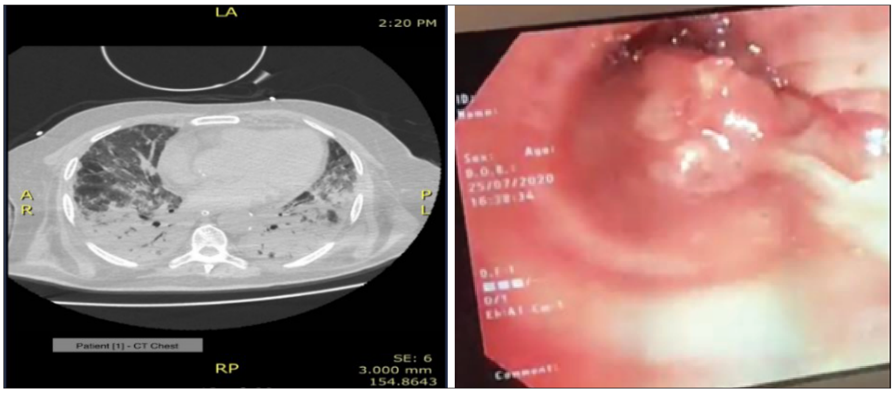

*Fig. 2. Computed tomography scan (A) for patient 1 and (B) fibrinous plugs in lobar bronchi.*

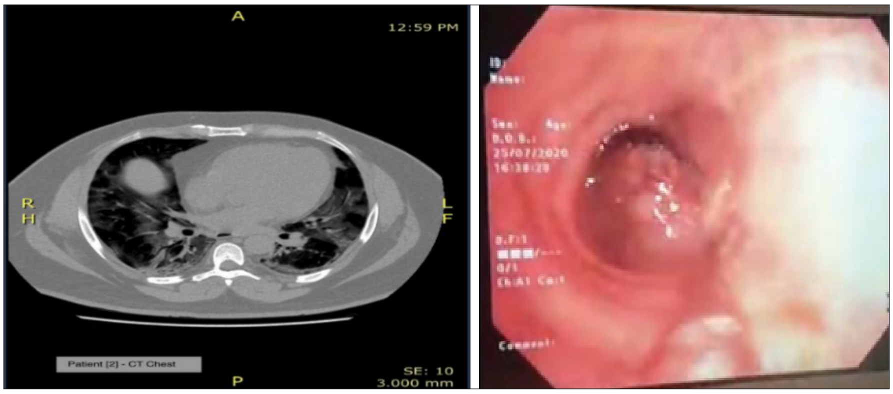

*Fig. 3. Computed tomography (A) scan for patient 2 and fibrinous plugs in subsegmental bronchi.*

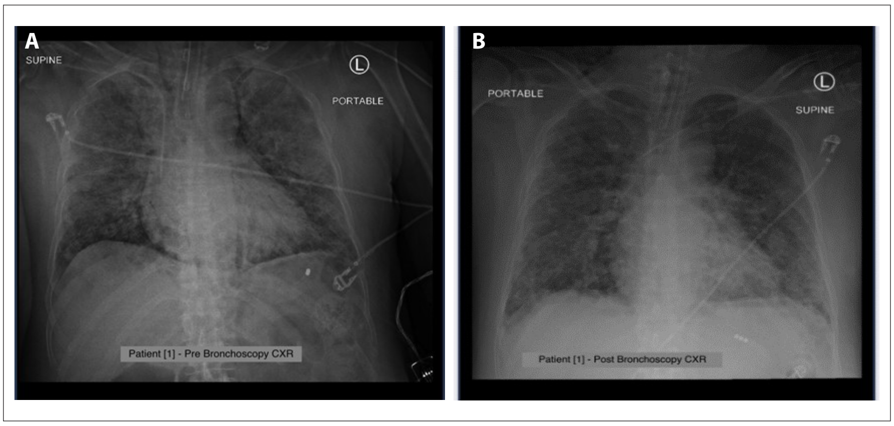

*Fig. 4. Chest radiograph for patient 1 (A) pre- and (B) post-bronchoscopy.*

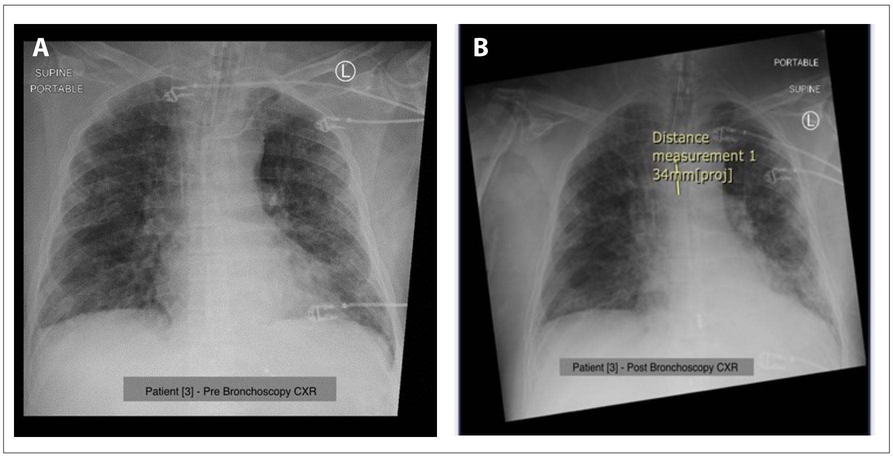

*Fig. 5. Chest radiograph for patient 3 (A) pre-and (B) post-bronchoscopy.*

**Table 2. Arterial blood gas pre- and post-bronchoscopy**

| Patient number | Day of ventilation | pH | | $pO_2$ (mmHg) | | $pCO_2$ (mmHg) | | $O_2$ saturation (%) | | P/F ratio | | MAP ($cmH_2O$) | | OI | |
|---|---|---|---|---|---|---|---|---|---|---|---|---|---|---|---|
| | | Pre 1 hr | Post 2 hr | Pre 1 hr | Post 2 hr | Pre 1 hr | Post 2 hr | Pre 1 hr | Post 2 hr | Pre 1 hr | Post 2 hr | Pre 1 hr | Post 2 hr | Pre 1 hr | Post 2 hr |
| 1 | 8 | 7.52 | 7.30 | 33 | 73.1 | 35 | 57.3 | 74 | 92 | 33 | 73 | 44 | 28 | | |
| 2 | 2 | 7.42 | 7.51 | 73.8 | 77.6 | 51.6 | 39.6 | 93 | 95 | 105 | 129 | 44 | 30 | | |
| 3 | 8 | 7.49 | 7.47 | 49 | 100 | 43.1 | 43 | 88 | 96 | 49 | 143 | 40 | 21 | | |
| 4 | 1 | 7.34 | 7.32 | 58.9 | 85.9 | 44.0 | 39.8 | 89 | 96 | 58.9 | 107 | 32 | 28 | | |
| 5 | 2 | 7.33 | 7.38 | 83.1 | 98.1 | 43.9 | 41.8 | 96 | 97 | 98 | 131 | 18 | 14 | | |
| 6 | 6 | 7.40 | 7.42 | 56 | 54 | 59 | 63 | 89 | 94 | 62 | 60 | 34 | 28 | | |
| 7 | 7 | 7.42 | 7.47 | 54 | 140 | 63 | 30 | 93 | 99 | 60 | 233 | 36 | 24 | | |
| 8 | 2 | 7.45 | 7.49 | 56 | 64 | 50 | 37 | 86.6 | 94.3 | 80 | 91 | 24 | 20 | 30 | 22 |
| 9 | 2 | 7.48 | 7.32 | 70 | 77 | 28 | 50 | 94 | 97 | 101 | 128 | 22 | 18 | 21 | 14 |
| 10 | 9 | 7.43 | 7.53 | 67 | 78 | 37 | 31 | 92.2 | 95.9 | 84 | 130 | 34 | 24 | 50.1 | 18 |
| 11 | 5 | 7.46 | 7.44 | 55 | 72 | 45 | 46 | 88.3 | 94 | 121 | 120 | 34 | 20 | 37.8 | 17 |
| 12 | 9 | 7.44 | 7.46 | 86 | 86 | 45 | 45 | 94.2 | 94.2 | 160 | 194 | 14 | 12 | 8.7 | 6.7 |
| 13 | 12 | 7.46 | 7.51 | 55 | 71 | 37 | 39 | 85.2 | 92.6 | 58 | 74 | 24 | 20 | 41 | 26 |
| 14 | 6 | 7.5 | 7.47 | 63 | 60 | 34 | 41 | 92.7 | 90.7 | 63 | 60 | 22 | 22 | 35 | 34 |

P/F ratio = Horowitz index for lung function; MAP = mean airway pressure; OI = oxygenation index.

the mucus within the endotracheal tube. The total time for the procedure for patient 1 was 3 hours, with the lowest oxygen saturation recorded at 23% for 30 seconds. For patient 3, the procedure time was 2.5 hours with the lowest oxygen saturation recorded at 40% for 40 seconds and the procedure time for patient 11 was 55 minutes with the lowest oxygen saturation recorded at 36% for 24 seconds

Patients 3, 5, 7 and 11 were extubated 72 hours after FFB to high -low nasal cannula and patients 1, 4, 6 and 9 are currently on minimal ventilator settings. The remainder of the patients were still on mechanical ventilation with $FiO_2$ >0.5 at the time of writing this report.

## Discussion

We have demonstrated that some patients with severe COVID-19 pneumonia and persistent hypoxaemia despite resolution of inflammatory parameters may respond to FFB following removal of mucus plugs. Although patients have been classified into H- and L-types, it does appear that those who require prolonged ventilation present and behave in a similar manner to patients with classical ARDS.[10] Some patients fail to improve their oxygenation despite optimal mechanical ventilation and pharmacotherapy inclusive of corticosteroids. These patients have a prolonged ventilatory course often complicated by secondary hospital-acquired sepsis with an associated high mortality.[7] Most international thoracic societies do not recommend therapeutic bronchoscopy except for control of pulmonary haemorrhage or for selected patients with lung atelectasis. However, our study demonstrated that radiological changes may be insensitive for the detection of significant mucus plugging and atelectasis may be missed. It is likely that at least some of the ground glass alveolar infiltrates observed in COVID-19 patients may represent filling of the alveolar spaces by mucus with or without some degree of segmental atelectasis and may also be a factor involved even in those with comorbidities predicting a worse outcome.

A study by Torrego *et al.*[11] confirmed the presence of mucus in the airways during bronchoscopy in 95% of 101 COVID-19 patients with an average ventilation duration of 6.6 days. Importantly, Earhart *et al.*[12] demonstrated that the use of the mucolytic dornase alfa in patients with COVID-19 improved outcomes and shortened duration of ventilation. A more recent randomised clinical trial in COVID-19 patients that received the oral mucolytic, bromhexine, showed that the benefit of bromhexine is maximised if started early and also showed that it can reduce respiratory symptoms, the need for ICU admission, intubation and mechanical ventilation, and mortality[13]

In our opinion, therapeutic FFB should be considered as an adjunctive therapy for COVID-19 patients with refractory hypoxaemia or even as a routine therapy around day 7 of mechanical ventilation if patients are slow to improve. It is critical that if hypoxaemia occurs during the procedure, oxygen delivery is maintained as patients appear to be protected from the effect of hypoxaemia so long as cardiac output and haemoglobin are maintained at the time of desaturation.[14,15] Therapeutic FFB to remove mucus plugs may be lifesaving and may reduce ventilator days and even mortality. We suggest that the routine use of mucolytics and thereafter bronchoscopy should be considered as rescue therapy before embarking on the use of ECMO. FFB is cheap, less invasive, and less complicated than ECMO. Airway obstruction by mucus plugs should be considered as an alternative explanation to the H-type phenotype or lung fibrosis in some patients and perhaps an additional pathophysiological phenotype should be included, the mucus type (M-type).

**Declaration.** None.

**Acknowledgements.** We would like to acknowledge Dr Karl Botman, Dr Lee-Anne Godinho and Dr Cornel Engelbrecht from the bronchoscopy team. We would also like to thank Fameesh Alunkadavath, Yvette Marais, Tsholofelu Boshomane from the COVID-19 Intensive Care Unit team and all the staff who were involved in the management COVID-19 patients. Finally, we would like to acknowledge Motheo Phalatse-Taban, Priya Pillay and Monique Berg from the physiotherapy team and Dr Avril Moodley, for editing the manuscript.

**Author contributions.** ET wrote the protocol, collected and analysed the data and wrote the manuscript. GR edited the protocol and the manuscript. Both authors approved the final manuscript.

**Funding.** None.

**Conflicts of interest.** None.

1. European Centre for Disease Prevention and Control. Communicable disease threats report, 4 March - 10 March 2012, week 10. https://www.ecdc.europa.eu/en/publications-data/communicable-disease-threats-report-4-march-10-march-2012-week-10 (accessed 4 August 2020).
2. To KK, Tsang OT, Leung WS, et al. Temporal profiles of viral load in posterior oropharyngeal saliva samples and serum antibody responses during infection by SARS-CoV-2: An observational cohort study. Lancet Infect Dis 2020;20: 565-574. https://doi.org/10.1016/ S1473-3099(20)30196-1
3. Ong SW, Tan YK, Chia PY, et al. Air, surface environmental and personal protective equipment contamination by severe acute respiratory syndrome coronavirus 2 (SARS-CoV-2) from a symptomatic patient. JAMA 2020;323(16):1610-1612. https://doi.org/10.1001/jama.2020.3227
4. Gattinoni L, Chiumello D, Caironi P, et al. COVID-19 pneumonia: Different respiratory treatment for different phenotypes? Intensive Care Med 2020;46(6):1099-1102. https://doi.org/10.1007/s00134-020-06033-2
5. Alhazzani W, Møller MH, Arabi YM, et al. Surviving sepsis campaign: Guidelines on the management of critically ill adults with coronavirus disease 2019 (COVID-19). Intensive Care Med 2020;28:1-34. https://doi.org/10.1007/s00134-020-06022-5
6. Sukhal S, Sethi J, Ganesh M, Villablanca PA, Malhotra AK, Ramakrishna H. Extracorporeal membrane oxygenation in severe influenza infection with respiratory failure: A systematic review and meta-analysis. Ann Card Anaesth 2017;20:14-21. https://doi.org/10.4103/0971-9784.197820
7. Varia M, Wilson S, Sarwal S, et al. Investigation of a nosocomial outbreak of severe acute respiratory syndrome (SARS) in Toronto, Canada. CMAJ 2003;169(4):285-292.
8. McGonagle D, O'Donnell JS, Sharif K, Emery P, Bridgewood C. Immune mechanisms of pulmonary intravascular coagulopathy in COVID-19 pneumonia. Lancet Rheumatol 2020;2(7):E437-E445. https://doi.org/10.1016/ S2665-9913(20)30121-1
9. Hare S, Rodrigues J, Nair A, et al. The continuing evolution of COVID-19 imaging pathways in the UK: A British Society of Thoracic Imaging expert reference group update. Clin Radiol 2020;75(6):399-404. https://doi.org/10.1016/j.crad.2020.04.002
10. Ferrando C, Suarez-Sipmann F, Mellado-Artigas R, et al. Clinical features, ventilatory management and outcome of ARDS caused by COVID-19 are similar to other causes of ARDS. Intensive Care Med 2020;24(5):367-368. https://doi.org/10.1007/s00134-020-06192-2
11. Torrego A, Pajares V, Fernandez-Arias C, Vera A, Mancebo J. Respiratory mechanics of COVID-19 versus non-COVID-19-associated acute respiratory distress syndrome. Am J Respir Crit Care Med 2020;202(2):284-287. https://doi.org/10.1164/rccm.202004-0945LE
12. Earhart AP, Holliday ZM, Hofmann HV, Schrum AG. Consideration of dornase alfa for the treatment of severe COVID-19 acute respiratory distress syndrome. New Microbes New Infect 2020;35:100689. https://doi.org/10.1016/j.nmni.2020.100689
13. Ansarin K, Tolouian R, Ardalan M, et al. Effect of bromhexine on clinical outcomes and mortality in COVID-19 patients. Bioimpacts 2020;10(4),209-215. https://doi.org/10.34172/bi.2020.27
14. Grocott MPW, Martin DS, Levett DZH, McMorrow RJ, Montgomery HE. Arterial blood gases and oxygen content in climbers on Mount Everest. New Eng J Med 2020;8;360(2):140-149. https://doi.org/10.1056/NEJMoa0801581
15. He HW, Liu DW, Long Y, Wang XT. High central venous-to-arterial CO2 difference/arterial-central venous O2 difference ratio is associated with poor lactate clearance in septic patients after resuscitation. J Crit Care 2016;31(1):76-81. https://doi.org/10.1016/j.jcrc.2015.10.017.

*Accepted 20 September 2020.*
